# The mucin-degradation strategy of *Ruminococcus gnavus*: The importance of intramolecular *trans*-sialidases

**DOI:** 10.1080/19490976.2016.1186334

**Published:** 2016-05-25

**Authors:** Emmanuelle H. Crost, Louise E. Tailford, Marie Monestier, David Swarbreck, Bernard Henrissat, Lisa C. Crossman, Nathalie Juge

**Affiliations:** a Institute of Food Research, The Gut Health and Food Safety Institute Strategic Program, Norwich Research Park, Norwich, United Kingdom; b The Genome Analysis Center, Norwich Research Park, Norwich, United Kingdom; c Architecture et Fonction des Macromolécules Biologiques, Université Aix-Marseille, CNRS UMR 7257, France; d Department of Cellular and Molecular Medicine, Faculty of Health and Medical Sciences, University of Copenhagen, Denmark; e School of Biological Sciences, University of East Anglia, Norwich, United Kingdom; f SequenceAnalysis.co.uk, NRP Innovation Center, Norwich, United Kingdom

**Keywords:** gut bacteria, glycoside hydrolase, intestinal mucin, intramolecular *trans*-sialidase, mucin glycans, *Ruminococcus gnavus*, sialic acid

## Abstract

We previously identified and characterized an intramolecular *trans*-sialidase (IT-sialidase) in the gut symbiont *Ruminococcus gnavus* ATCC 29149, which is associated to the ability of the strain to grow on mucins. In this work we have obtained and analyzed the draft genome sequence of another *R. gnavus* mucin-degrader, ATCC 35913, isolated from a healthy individual. Transcriptomics analyses of both ATCC 29149 and ATCC 35913 strains confirmed that the strategy utilized by *R. gnavus* for mucin-degradation is focused on the utilization of terminal mucin glycans. *R. gnavus* ATCC 35913 also encodes a predicted IT-sialidase and harbors a Nan cluster dedicated to sialic acid utilization. We showed that the Nan cluster was upregulated when the strains were grown in presence of mucin. In addition we demonstrated that both *R. gnavus* strains were able to grow on 2,7-anyhydro-Neu5Ac, the IT-sialidase transglycosylation product, as a sole carbon source. Taken together these data further support the hypothesis that IT-sialidase expressing gut microbes, provide commensal bacteria such as *R. gnavus* with a nutritional competitive advantage, by accessing and transforming a source of nutrient to their own benefit.

## Introduction

The gastrointestinal (GI) tract is inhabited by a diverse microbial community (microbiota) that influences host health through a number of mechanisms, including the production of metabolites, protection against pathogens, and interactions with the host immune system and physiology. The microbiota composition varies longitudinally along the GI tract but also transversally from the mucosa to the lumen.^1^ In the large intestine, the mucus shows a bi-layer organization, with the outer layer providing a habitat to the bacteria whereas the inner layer keeps them away from the epithelium surface.^2^ Mucins facilitate microbial tropism through the presentation of glycans which may impact colonization.^3^ The terminal mucin O-glycans have been proposed to serve as metabolic substrates, providing a nutritional advantage to bacteria which have adapted to the GI mucosal environment.^4,5^ Given the diversity and complexity of intestinal mucin glycan structures, strategies for deconstructing these molecules rely on the cooperative action of a number of proteases, sulfatases, and glycosidases encoded by the genome of mucin-degrading bacteria. So far, only a limited number of bacterial species/strains from the Bacteroidetes, Firmicutes, Actinobacteria, and Verrucomicrobia phyla have been studied for their ability to consume mucins and it is becoming apparent that the strategy used by these mucin-degraders is strain and species-dependent.^5^ Based on these studies, a number of glycoside hydrolases (GH) families as classified in the CAZy database (www.cazy.org)^6^ have been involved in mucin-degradation.^5^ These include neuraminidases/sialidases (GH33), fucosidases (GH29 and GH95), exo- and endo-β-N-acetylglucosaminidases (GH84 and GH85), β-galactosidases (GH2, GH20 and GH42), α-N-acetylglucosaminidases (GH89), endo-β1–4-galactosidases (GH98) and α-N-acetylgalactosaminidases (GH101, GH129) (www.cazy.org). Most bacteria appear to have incomplete packages of GHs for utilizing host mucins and so degradation of mucin in the human gut is likely to be carried out by a consortium of bacteria.

*Ruminococcus gnavus* is a prominent member of the gut microbial community,^7,8^ belonging to the Firmicutes division, Clostridia class and XIVa cluster, Lachnospiraceae family.^9^ In our previous work, we showed that the ability of *R. gnavus* to utilize mucins was strain-dependent and associated with sialic acid metabolism although *R. gnavus* is not able to grow on free sialic acid (Neu5Ac).^10^ We further showed that the mucin-degrader *R. gnavus* ATCC 29149 strain produces an intramolecular *trans*-sialidase (IT-sialidase) that cleaves off terminal α2–3-linked sialic acid from glycoproteins, releasing 2,7-anhydro-Neu5Ac instead of sialic acid,^11^ suggesting a novel mechanism of mucosal adaptation. Here we further explored the mucin-degradation strategy of *R. gnavus* by expanding the work to another mucin-degrading strain, *R. gnavus* ATCC 35913, for which we obtained the genome sequence, and carried out comparative sequence and transcriptomics analyses and confirmed the ability of the strains to grow on 2,7-anhydro-Neu5Ac, supporting the role of IT-sialidase in the mucin-degrading strategy utilized by *R. gnavus*.

## Materials and methods

### Materials

D-glucose (Glc), N-acetylneuraminic acid (Neu5Ac), 2′-(4-Methylumbelliferyl)-α-D-*N*-acetylneuraminic acid (4MU-Neu5Ac) and type III pig gastric mucin (PGM) were purchased from Sigma-Aldrich (St Louis, MO).^10^ Purified pig gastric mucin (pPGM) was obtained as previously described.^12^ 6′-sialyllactose (6′SL) was kindly provided by Glycom A/S (Lyngby, Denmark). 3′-sialyllactose (3′SL) was purchased from Carbosynth Limited (Campton, UK).

### Bacterial strains and growth conditions

*R. gnavus* strains were routinely grown in an anaerobic cabinet (Don Whitley, Shipley, UK) in BHI-YH as previously described.^10^ Growth on single carbon sources utilized anaerobic basal YCFA medium^13^ supplemented with 27.7 mM of specific mono- or oligosaccharides as indicated or 1% (wt/vol) pPGM. The growth assays were performed in 96-well plates with 200 μL of medium/well with a single point OD measurement at 595 nm after 44 hours. When the bacteria were grown with Glc or pPGM as sole carbon source with sampling for RNA extraction, the culture was performed in 14 mL tubes. Growth was determined spectrophotometrically by monitoring changes in optical density at 600 nm compared to the same medium without bacterium (Δ OD600 nm) hourly for 9 hours.

### Genomic DNA extraction from R. gnavus ATCC 35913 and sequencing

For the isolation of *R. gnavus* ATCC 35913-chromosomal DNA, cells from a 50 mL-overnight culture were harvested by centrifugation (10 000 g, 5 min, 4°C). The cell pellet was washed with 5 mL of TES buffer (10 mM Tris, 1 mM EDTA, 0.1 M NaCl, pH 8), resuspended in 5 mL of TES buffer supplemented with lysozyme (20 mg.mL^−1^) and incubated for 15 min at 37°C. Then, complete lysis was achieved by addition of 1 mL of 20% sodium dodecyl sulfate (SDS) and incubation for 10 min at 50°C. The mixture was then extracted by three consecutive treatments: first, with 5 mL of phenol pH 7.9 then with 5 mL of phenol-chloroform-isoamyl alcohol (25:24:1) and finally with 5 mL of chloroform-isoamyl alcohol (24:1). After precipitation with cold absolute ethanol, the genomic DNA was resuspended in 2 mL of TE buffer (10 mM Tris, 1 mM EDTA, pH 8). Traces of RNA were removed by a treatment with RNAse ONE (Promega, Madison, WI) used as recommended by the manufacturer. The DNA was again precipitated with 0.3 M sodium acetate (pH 5.2) and 70% ice-cold ethanol. Finally, it was dissolved in 1.5 mL of TE. Quality and quantity were assessed using NanoDrop 1000 UV-Vis Spectrophotometer and by electrophoresis on 0.7% agarose gel. The DNA was sequenced on the Roche 454 GS Flx platform using 500 ng of input DNA. The sequencing library was produced by the rapid library and emulsion PCR method and run on a single 454 picotiter plate at The Genome Analysis Center (TGAC). The 454 reads were assembled with Newbler (Roche) and annotated at first using Rast (NMPDR)^14^ with specific additions to the annotation from the authors, including some manual curation. The EMBL accession numbers of the annotated contigs are FCFA01000001-FCFA01000241.

### Comparative CAZome analysis

The translated protein sequences of *R. gnavus* E1, ATCC 29149 and ATCC 35913 were compared to the full length sequences derived from the Carbohydrate-Active enZymes (CAZy) database (www.cazy.org)^15^ using BLAST.^16^ The sequences that had an e-value >0.1 were assigned to glycoside hydrolases (GH), glycoside transferases (GT), polysaccharide lyases (PL), carbohydrate esterases (CE), and carbohydrate binding module (CBM) families using a parallel procedure involving a BLAST search against partial sequences corresponding to individual GH, GT, PL, CE and CBM modules and a HMMer search^17^ using hidden Markov models built for each CAZy module family.^15^ The counts for each CAZy family of each strain were then compared and the putative function of the proteins of interest was evaluated by alignment with the sequences of biochemically characterized enzymes.^14^

### Total RNA extraction from R. gnavus strains

Total RNA was extracted from 10 mL of mid- to late exponential phase cultures of ATCC 29149 and ATCC 35913 in YCFA supplemented with one carbon source (Glc or pPGM). Four biological replicates were performed for each carbon source. The RNA was stabilized prior to extraction by adding 1/5 vol of phenol (pH 4.3) : ethanol (1:9) mixture to 1 vol of culture then incubating 30 min on ice and finally pelleting the cells for 5 min at 10,000 g at 4°C. Cell pellets were stored at −80°C before extraction. Extraction was performed using a traditional method using phenol and chloroform. Genomic DNA contamination was removed by DNAse treatment using TURBO DNA-free kit (Life Technologies Ltd, Paisley, UK) according to supplier's recommendations. The purity, quantity and integrity of the extracted RNA were assessed before and after DNase treatment, with NanoDrop 1000 UV-Vis Spectrophotometer (Thermo Fischer Scientific, Wilmington, DE) and with Agilent RNA 600 Nano kit on Agilent 2100 Bioanalyzer (Agilent Technologies, Stockport, UK).

### RNA sequencing

Transcriptome libraries were constructed using the Illumina TruSeq RNA sample preparation kit with modifications. The rRNA was depleted using Ribo-Zero™ rRNA Removal Kit for Gram-Positive Bacteria (Epicentre Illumina). The rRNA removal was confirmed with a Pico chip run on Bioanalyzer 2100 (Agilent) and the quantity measured with the Qubit RNA kit and Qubit fluorometer (Invitrogen). The resulting ribosomal depleted RNA was then fragmented for 8 min at 94°C using the Elute, Fragment, Prime buffer from Illumina TruSeq RNA kit. These conditions give final libraries of around 400 bp. The samples were then processed following the standard TruSeq RNA protocol.

The 4 Illumina libraries were normalized and equimolar pooled to 15 nM using elution buffer (Qiagen). The library pool was then diluted to 2 nM with NaOH and 5 μL transferred into 995 μL HT1 (Illumina) to give a final concentration of 10 pM. A portion (120 μL) of the diluted library pool was then transferred into a 200 μL strip tube, spiked with 1% PhiX Control v3 and placed on ice before loading onto the Illumina cBot. The flow cell was clustered using TruSeq Single End Cluster Generation Kit v3, following the Illumina SE_amplification_Linearization_Blocking_PrimerHyb_v8 recipe. Following the clustering procedure, the flow cell was loaded onto the Illumina HiSeq2000 instrument following the manufacturer's instructions. The sequencing chemistry used was TruSeq SBS Kit v3-HS using HiSeq Control Software 1.4.8 and RTA 1.12.4.2. The library pool was run in a single lane for 100 cycles. Reads in bcl format were demultiplexed based on the 6 bp Illumina index by CASAVA 1.8, allowing for a one base-pair mismatch per library, and converted to FASTQ format by bcl2fastq.

rRNA depletion levels were assessed following read mapping. To carry this out, rRNA genes were initially manually curated on the ATCC 29149 genome. To compare the transcript expression levels across all samples, the RNA-seq reads were mapped onto the *R. gnavus* ATCC 29149 genome with the open source tool Bowtie v0.12.9^18^ using default parameters. Differentially expressed genes were identified using the DESeq2 R package^19^ with raw counts normalized to the effective library size. Results were also assessed using DeSeq R package,^20^ edgeR^21^ and simple RPKM normalization methods to check the DESeq2 analysis. A QC check was performed using a sample correlation matrix and plotting it as a tree. The significance of differential expression was determined by the Benjamini–Hochberg^22^ corrected p-values of the Wald test for the negative binomial between two conditions. The threshold for significance was set to p < 0.05 and a Log2 (fold change) >1. Error bars were taken as the standard error of the Log2 fold change.

### Synthesis and analysis of 2,7-anhydro-Neu5Ac

The synthesis of 2,7-anhydro-Neu5Ac was carried out using a modified method.^23^ Briefly, 4-Methylumbelliferyl (MU)-Neu5Ac was incubated with recombinant IT-sialidase^11^ in ammonium formate buffer (100 mM) at 37°C, pH 6.5 overnight. The reaction was terminated by the addition of an equal volume of ethanol and the precipitate removed by centrifugation (4000 g for 20 min). The supernatant was evaporated to dryness, dissolved in chloroform/methanol and subjected to Folch partitioning. Fractions containing 2,7-anhydro-Neu5Ac were identified by thin-layer chromatography (TLC) using silica gel 60 F254 (Merck) for the stationary phase and butanol/ acetic acid/ water at a ratio 2:1:1 for the mobile phase and vizualization by incubating the TLC into a potassium permanganate solution followed by heating. The purified product was analyzed by electrospray ionization mass spectrometry (ESI-MS) and H^1^ NMR. MS spectra were acquired on Expression CMS (Advion) with ESI ionization using direct injection operated in a negative ion mode. Advion Data Express (version 2.2.29.2) software package was used to evaluate the MS data. The ^1^H NMR spectrum was recorded in D_2_O at 400 MHz on a Bruker Avance spectrometer (Bruker BioSpin GmbH, Rheinstetten, Germany) running Topspin 3.2 software, and was fitted with a cryoprobe. ^1^H NMR spectrum was acquired with 16 scans, a spectral width of 8,223.6 Hz, an acquisition time of 3.98 s and a relaxation delay of 4.0 s. The spectrum was transformed with a 0.3-Hz line broadening, manually phased, baseline corrected and referenced by setting the water signal to 4.7 p.p.m.

## Results and discussion

### R. gnavus ATCC 35913 grows on mucin and 3′-sialyllactose but not on sialic acid or 6′-sialyllactose

**Figure 1.**
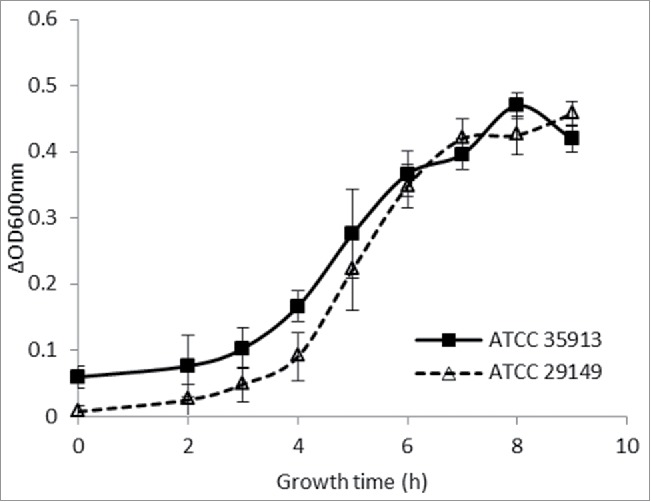
Growth curves of *R. gnavus* ATCC 29149 and ATCC 35913 on pPGM. The growth curves represent the average growth, measured at OD600nm, of at least 3 biological replicates.

Previous work showed that the ability of *R. gnavus* to utilize mucins is strain-dependent with ATCC 29149 but not E1 being able to grow on mucins.^10^ Here we tested the ability of *R. gnavus* ATCC 35913 to grow on mucin using purified porcine gastric mucin (pPGM) as source of carbon. *R. gnavus* ATCC 35913, has been isolated from fecal sample of a healthy human adult.^24^ Spectrophotometric measurements were made every hour for up to 9 hours (Fig. 1). The growth curves were analyzed using the in-house-developed DMFit program. For both strains the lag phase was just below 2 hours and the maximum ΔOD at 600 nm was identical. As previously reported for *R. gnavus* ATCC 29149,^10^ *R. gnavus* ATCC 35913 grew on Glc and on 3′-sialyllactose (Neu5Acα2–3Galβ1–4Glc, 3′SL) but was unable to grow in presence of sialic acid (Neu5Ac) or 6′-sialyllactose (Neu5Acα2–6Galβ1–4Glc, 6′SL) as sole carbon source (Table 1). These data suggest that the strategy used by the mucin-degrader *R. gnavus* ATCC 35913 is similar to that previously reported for ATCC 291495.^5,10^

**Table 1.** Growth assay of the 3 strains of *R. gnavus* with several carbon sources.

	Carbon source							
Strain	none	Glc	3′SL	6′SL	MU-Neu5Ac	MU	Neu5Ac	2,7-anhydro-Neu5Ac
E1	−	+	−	−	−	−	−	−
ATCC29149	−	+	+	−	+	−	−	+
ATCC35913	−	+	+	−	+	−	−	+

*Note*. +: growth; −: no growth; Glc was used as a positive control

Abbreviations: Glc, glucose; 3′SL, 3′-sialyllactose; 6′SL, 6′-sialyllactose; MU-Neu5Ac, 2′-(4-Methylumbelliferyl)-α-D-N-acetylneuraminic acid; MU, 4-Methylumbelliferol; Neu5Ac, N-acetylneuraminic acid; 2,7-anhydro-Neu5Ac, 2,7-anhydro-N-acetylneuraminic acid.

### In silico and transcriptomics analysis confirm IT-sialidase as a major determinant for R. gnavus mucin-degrader strains

**Figure 2.**
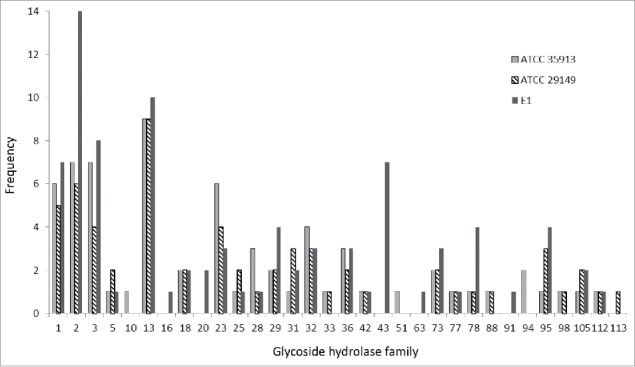
Comparison of the distribution of glycoside hydrolases (GHs) between *R. gnavus* strains. GHs are represented by light gray boxes for *R. gnavus* ATCC 35913, striped boxed for *R. gnavus* ATCC 29149 and dark gray boxes for *R. gnavus* E1.

In order to gain insights into the molecular basis of *R. gnavus* ATCC 35913 mucin-degradation, we sequenced and analyzed the *R. gnavus* ATCC 35913 genome and compared its mucin-degrading potential to that of *R. gnavus* ATCC 29149 and E1 strains. The draft assembly possessed 241 contigs of average length 15510 bp and median length of 1792 bp. The longest contig was 222692 bp, while the shortest contig was 102 bp. The *R. gnavus* ATCC 35913 genome is 3.74 Mb long with a GC content of 43%, which is similar to the GC% of E1 and ATCC 29149 (42.44% and 42.9% respectively). Genomic analysis identified 99 full length genes and 12 fragments of genes encoding CAZymes (www.cazy.org), corresponding to approximately 2.87% of CDS dedicated to carbohydrate metabolism. *R. gnavus* ATCC 35913 genome contains the following number of CAZyme modules; 36 GTs, 4 CEs, 1 PL, 12 CBMs and 67 GHs. Nine of the CAZy genes encode two modules, almost always a CBM and a GH, although one of the fragments contains two GH2 domains. There are also 4 predicted proteins solely composed of a CBM (2 CBM32, 1 CBM50 and 1 CBM13) with no predicted associated catalytic module. Most of *R. gnavus* ATCC 35913 CAZome is represented by genes encoding GHs distributed into 26 GH families. A comparison of *R. gnavus* ATCC 35913 repertoire of GH families with that of *R. gnavus* ATCC 29149 and E1 strains is presented in Figure 2. Both mucin-degrading strains follow a similar GH representation with most GH families being common between the two strains apart from GH10 (xylanase), GH51 (endoglucanase, xylanase, L-arabinofuranosidase) and GH94 (phosphorylase) being specific of *R. gnavus* ATCC 35913 and GH113 (β-mannanase) being specific for *R. gnavus* ATCC 29149. Among the GH families which members have been implicated in mucin-degradation^5^ are GH2 β-galactosidases, GH29 and GH95 fucosidases and a GH33 sialidase. Interestingly, the genome of *R. gnavus* ATCC 35913 encodes a predicted GH33 sialidase sharing 100% identity with the enzyme which was characterized as an IT-sialidase in ATCC 29149, and is absent in the non-degrading strain E1. Similarly GH98 which encodes a predicted endo-β-galactosidase appears to be specific for the *R. gnavus* mucin-degrading strains. In contrast, all of the *R. gnavus* strains (including the E1 strain^10^) contain multiple GH29 and GH95 α-fucosidases, GH2 β-galactosidases and a high proportion of GH13 members, which is the major GH family acting on substrates containing α-glucoside linkages; a detailed analysis of their substrate specificity is warranted to assess their contribution to the carbohydrate preference of each strain.

**Figure 3.**
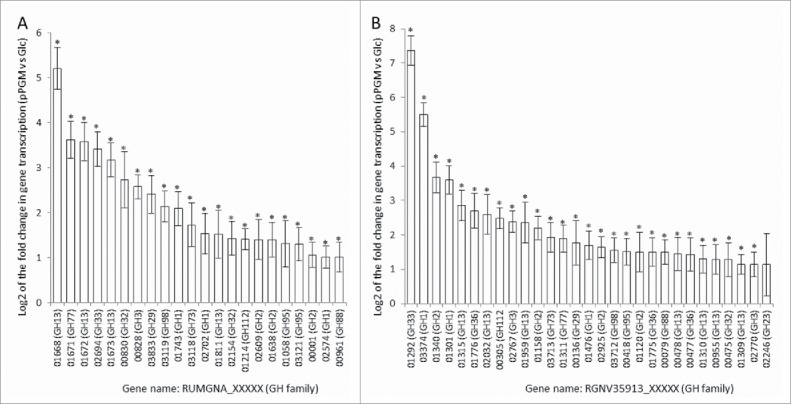
Relative level of transcription of GH genes in *R. gnavus* ATCC 29149 (A) and ATCC 35913 (B). The transcriptomic analysis has been performed by RNASeq from *R. gnavus* grown in presence of pPGM and compared to Glc as sole carbon source. The relative level of transcription was expressed as the Log2 of the fold change in gene transcription and the figures showed averages of 4 biological replicates for the GH genes that exhibited increased transcription (Log2 fold change > 1). Data were analyzed by DESeq2.^19^ The significance of differential expression was determined by the Benjamini-Hochberg^21^ corrected p-values of the Wald test of the negative binomial test per each set of two conditions. The transcription level was considered significantly increased when p < 0.05 and a Log2 (fold change) >1 and significant results were labeled with *. Error bars were plotted as the standard error of the Log2 fold change.

To further assess the importance of these GHs in the ability of the mucin-degrader strains to grow on mucins, we analyzed the transcriptional activity of ATCC 29149 (Fig. 3A) and ATCC 35913 (Fig. 3B) by RNAseq analysis. The level of expression was compared to a reference dataset of the strains grown in minimal medium with Glc as the sole carbon source. These data confirmed the increased transcription of the GH33 sialidase gene (*nanH*) when ATCC 29149 was grown with mucin, previously observed by qPCR, and are in agreement with the implication of this extracellular enzyme in enabling *R. gnavus* ATCC 29149 to grow on mucin.^10^ Similarly the gene encoding GH33 sialidase from *R. gnavus* ATCC 35913 was highly upregulated in presence of mucins, the highest of all GHs (Fig. 3B). The GH98 endo-β1–4-galactosidase gene expression was also induced in presence of mucin for both strains. Other mucin-specific upregulated genes included some GH29 and GH95 fucosidases (Fig. 3A and B). It is also worth noting that other GH families, not implicated in mucin-degradation, showed gene expression induction including GH13 and GH1 (mainly exo-acting enzymes cleaving a β linkage) family members, which raises questions on the substrate specificity of these enzymes.

### *R. gnavus* ATCC 35913 encodes a functional sialic acid utilization operon

**Figure 4.**
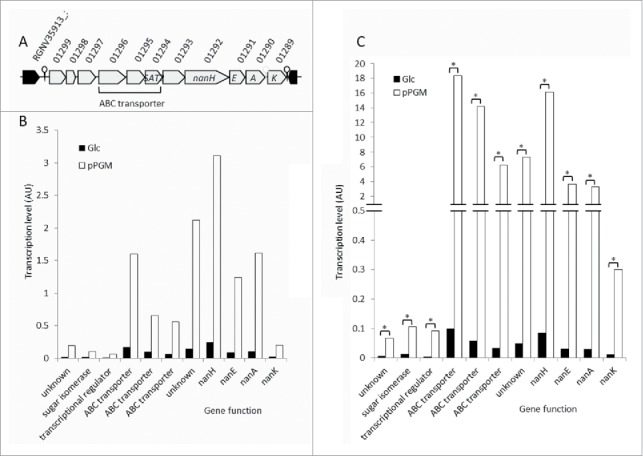
The Nan locus in *R. gnavus* ATCC 29149 and ATCC 35913. (A) Schematic representation of the *nan* genetic organization in ATCC 35913. RGNV35913_01299 encodes a putative GDSL-like protein. RGNV35913_01298encodes a putative sugar isomerase involved in sialic acid catabolism. RGNV35913_01297 encodes a protein with homology with transcriptional regulators of the AraC family. The following 3 genes code for a predicted solute-binding protein (RGNV35913_01296) and two putative permeases (RGNV35913_01295 and RGNV35913_01294), components of a sugar ABC transporter. The following gene has homology with oxidoreductases from the Gfo/Idh/MocA family. The sialidase gene *nanH* (RGNV35913_01292) predicted to encode the GH33 enzyme comes next. Then *nanE* (RGNV35913_01291), which encodes a predicted ManNAc-6-P epimerase is followed by *nanA* (RGNV35913_01290) encoding a putative Neu5Ac lyase. *nanK* (RGNV35913_01289) is the last gene of the cluster, coding for a predicted ManNAc kinase. The previously described *R. gnavus* ATCC 29149 nan cluster^10^ shares 99.9% identity with the one present in ATCC 35913. Level of transcription of *nan* genes in *R. gnavus* ATCC 29149 (B) or ATCC 35913 (C). *R. gnavus* was grown in basal YCFA medium supplemented with either glucose (Glc) or mucin (pPGM) as sole carbon source. Cells were collected during the exponential phase of growth; RNA was extracted from 4 biological replicates for each carbon sources. The level of transcription of each gene was determined by RNASeq. The transcription of each gene was compared when the bacterium grew with pPGM vs. Glc using the R package DESeqx; it was considered significantly increased when the transcript was present at least twice more frequently, with a padj value (p-value adjusted for multiple testing) <=0.05 (* padj<=0.05).

*R. gnavus* ATCC 29149 encodes an 11.7-kb Nan operon containing genes encoding putative proteins involved in the utilization of sialic acid whereas this cluster is absent in the non-mucin degrading strain *R. gnavus* E1.^10^ Bioinformatics analysis of the *R. gnavus* ATCC 35913 genome suggests the presence of an 11.7- kb Nan cluster sharing 99.9% identity with the one present in ATCC 29149 and constituted of 11 open reading frames (ORFs) (Fig. 4A). The first gene of the cluster (RGNV35913_01299) is predicted to code for a GDSL-like protein belonging to the SGNH-hydrolase family, a diverse family of esterases and lipases, which comprises for example mucin-desulfating sulfatases (N-acetylglucosamine-6-sulfatases). The second gene (RGNV35913_01298) encodes a putative sugar isomerase belonging to the YhcH/YjgK/YiaL family. The following one (RGNV35913_01297) encodes a protein with homology with transcriptional regulators of the AraC family. The following 3 genes code for a predicted solute-binding protein (RGNV35913_01296) and two putative permeases (RGNV35913_01295 and RGNV35913_01294), components of a sugar ABC transporter; RGNV35913_01294 has specific homology with putative sialic acid transporters of the SAT2 family. The following gene has homology with an oxidoreductase from the Gfo/Idh/MocA family. The sialidase gene *nanH* (RGNV35913_01292) predicted to encode the GH33 enzyme comes next. Then *nanE* (RGNV35913_01291), which encodes a predicted N-acetylmannosamine-6P (ManNAc-6-P) epimerase converting ManNAc-6-P into N-acetylglucosamine-6-P (GlcNAc-6-P), followed by *nanA* (RGNV35913_01290) encoding a putative Neu5Ac lyase involved in the breaking down of Neu5Ac into ManNAc and pyruvate. *nanK* (RGNV35913_01289) is the last gene of the cluster, coding for a predicted ManNAc kinase, involved in the phosphorylation of ManNAc into ManNAc-6-P.

Previous data showed that the transcription of *nanH* was up-regulated when *R. gnavus* ATCC 29149 was grown in presence of mucin, as determined by microarrays and qPCR.^10^ Here we analyzed the transcriptional activity of this cluster by RNAseq analysis in both *R. gnavus* mucin-degrader strains grown on mucin as sole carbon source. We confirmed that all the genes involved in the release and transport of sialic acid -* nanH* and the 3 genes coding for an ABC transporter - as well as all the genes putatively involved in sialic acid metabolism inside the cell- were upregulated when *R. gnavus* ATCC 29149 and ATCC 35913 were grown with mucin (Fig. 4B and C). The average transcription of all the genes of the cluster (including *nanA, nanE* and *nanK*) was increased by at least 7 times when ATCC 29149 grew with pPGM as compared to Glc. Similarly, the change in transcription of the *nan* genes in ATCC 35913 is at least 8-fold and this increase was statistically significant for all 11 genes of the operon (Fig. 4C). These results indicate that these strains have adapted to scavenge sialic acid from host sialoglycans.

### *R. gnavus* ATCC 29149 and ATCC 35913 grow on IT-sialidase transglycosylation product

In order to test the ability of *R. gnavus* ATCC 29149 and ATCC 35913 strains to utilize 2,7-anydro-Neu5Ac as a carbon source, we first performed the *in vitro* enzymatic synthesis of the transglycosylation product (not commercially available). The recombinant IT-sialidase from *R. gnavus* ATCC 29149 heterologously expressed in *E. coli*^11^ was used in presence of MU-Neu5Ac to produce 2,7-anhydro-Neu5Ac in amounts enabling screening the growth of *R. gnavus* strains in microtiter plates. The obtained product 2,7-anhydro-Neu5Ac was ∼80% pure according to ESI-MS and H^1^ NMR analysis (Fig. S1). The by-product of the reaction, identified as MU, did not interfere with the cell growth (data not shown). We showed that both *R. gnavus* ATCC 35913 and ATCC 29149 could grow on MU-Neu5Ac or 2,7-anhydro-Neu5Ac as sole carbon source, while the strains were unable to grow on Neu5Ac or MU. It is worth noting that E1 was unable to grow on any of these carbon sources (Table 1).

**Figure 5.**
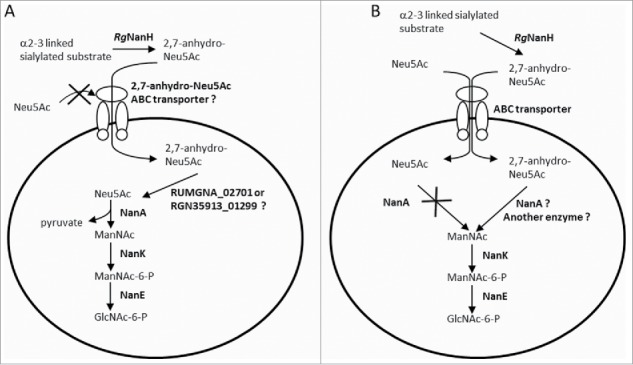
Proposed pathways for the catabolism of sialic acid in *R. gnavus* ATCC 29149 and ATCC 35913. *Rg*NanH releases 2,7-anhydro-Neu5Ac from α2–3 linked sialylated substrates. (A) It can be hypothesized that 2,7-anhydro-Neu5Ac is transported inside the bacterium *via* a 2,7-anhydro-Neu5Ac-specific ABC transporter composed of a solute-binding protein (RUMGNA_02698 in ATCC 29149;RGNV35913_01296 in ATCC 35913) and two putative permeases (RUMGNA_02697 and RUMGNA_02696 in ATCC 29149; RGNV35913_01295 and RGNV35913_01294 in ATCC 35913) and then hydrolyzed into Neu5Ac, possibly by the action of RUMGNA_02701 or RGNV35913_01299, before being catabolized into GlcNAc-6-P following the traditional pathway by the successive action of NanA (Neu5Ac lyase), NanK (ManNAc kinase) and NanE (ManNAc-6-P epimerase). (B) Alternatively, both 2,7-anhydro-Neu5Ac and Neu5Ac could enter the cells *via* the ABC transporter but NanA would either be inactive or specific for 2,7-anhydro-Neu5Ac, explaining the absence of growth of the bacteria on sialic acid.

Taken together we propose a model whereby 2,7-anhydro-Neu5Ac is transported into the bacteria *via* the ABC transporter composed of a solute-binding protein (RUMGNA_02698 or RGNV35913_01296) and two putative permeases (RUMGNA_02697 and RUMGNA_02696, or RGNV35913_01295 and RGNV35913_01294) (Fig. 5A). It is not known whether this transporter is specific of the 2,7-anhydro-Neu5Ac or could also transport Neu5Ac. Once inside the cell, 2,7-anhydro-Neu5Ac could be hydrolyzed into Neu5Ac by the product of RUMGNA_02701 or RGNV35913_01299, which encodes a putative protein belonging to the GDSL-like family, a diverse family of esterases and lipases. This family contains SGNH-hydrolases, a sub-family of enzymes with multifunctional properties such as broad substrate specificity and regiospecificity, some of them have been shown to act as esterase on acetylated sugar e.g. NanS, an enzyme able to deacetylate the 9-O-acetyl Neu5Ac into Neu5Ac.^25^ RUMGNA_02701 or RGNV35913_01299 predicted protein possesses the four strictly conserved amino acids, Ser-Gly-Asn-His, involved in the catalytic function of these enzymes as well an Asp residue upstream of His, another feature of these enzymes.^26^ Neu5Ac would then be catabolized into GlcNAc-6-P by the successive action of NanA (Neu5Ac lyase), NanK (ManNAc kinase) and NanE (ManNAc-6-P epimerase) before entering the glycolytic pathway, in accordance with the predicted specificities of these enzymes.^27^ Alternatively it can be hypothesized that NanA shows specificity for 2,7-anhydro-Neu5Ac instead of Neu5Ac (Fig. 5B).

Gaining mechanistic insights into the strategies used by members of the gut microbiota to utilize mucin glycans is important to understand how the mucus-associated ecosystem is maintained. Due to their proximity to the host immune system, mucus-associated microorganisms may have a disproportionate impact on health.^28^ Several lines of evidence indicate that mucin-degrading bacteria such as *R. gnavus* may play an important role in dysbiosis by increasing total mucosa-associated bacteria in inflammatory bowel diseases.^29^ Furthermore *R. gnavus* is involved iso-bile acid (BA) biosynthesis,^30,31^ leading to the production of secondary BAs which play a role in modulating gut community composition.^32^ Identification of keystone and foundation taxa in the mucus-associated GI microbiota is important for novel diagnostic strategies and therapeutic modulation.^33^ Keystone species of the GI microbiota have been identified for bacteria that are instrumental to the degradation of dietary resistant starch.^34^ With regards to host mucin degradation, we believe that the presence of IT-sialidases will provide gut microbes such as *R. gnavus* with a competitive nutritional advantage, allowing the bacteria to thrive within mucosal environments by scavenging sialic acid from host mucus in a form, 2,7-anhydro-Neu5Ac, that can be used to their own benefit. Furthermore, once the terminal sugars and blood group antigens are removed and used to support growth of *R. gnavus*, the mucin core glycans are exposed to enzymatic degradation by other members of the gut microbiota, thus favoring metabolic cross-feeding within this mucosal niche. This dual strategy may fit the representation of *R. gnavus* as a keystone member of the mucus-associated gut microbiota.

## Supplementary Material

KGMI_A_1186334_Figure_S1.png

## Abbreviations

CAZyme: carbohydrate-active enzyme
CBM: carbohydrate binding module
CDS: coding DNA sequence
GH: glycoside hydrolase
GI: gastrointestinal
Glc: glucose
IT-sialidase: intramolecular *trans*-sialidase
MU: 4-methylumbelliferyl
Neu5Ac: N-acetylneuraminic acid
ORF: open reading frame
Ppgm: purified pig gastric mucin
SL: sialyllactose
