# Supplementary figures and images for: The mucin-degradation strategy of Ruminococcus gnavus: The importance of intramolecular trans-sialidases
Source: Gut Microbes. 2016 May 25;7(4):302–12. doi: 10.1080/19490976.2016.1186334 (PMC4988440; doi:10.1080/19490976.2016.1186334)

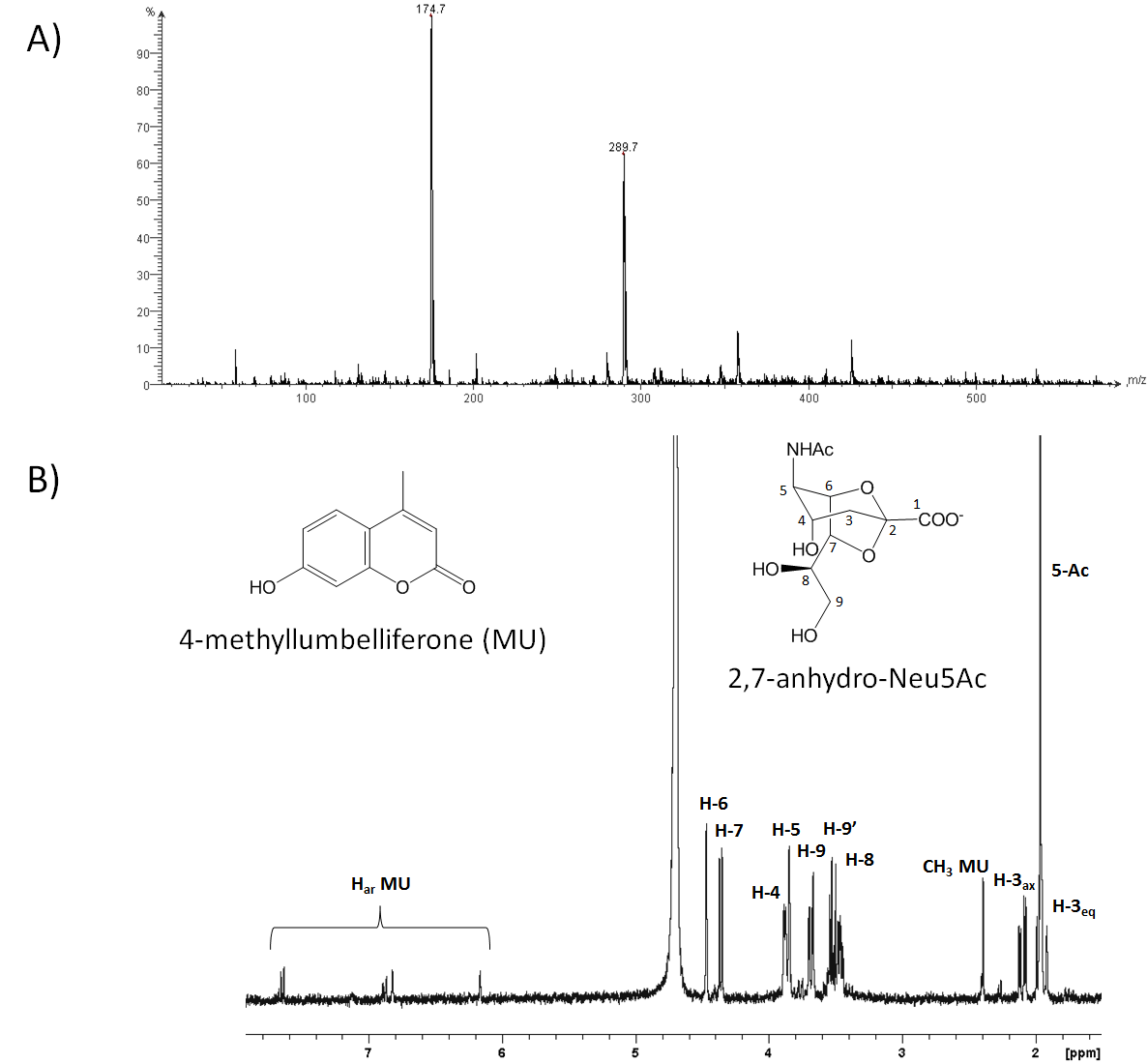

Supplement: KGMI_A_1186334_Figure_S1.png [file kgmi-07-04-1186334-s001.png]
